# Differential effects of distance decay on hospital inpatient visits among subpopulations in Florida, USA

**DOI:** 10.1007/s10661-019-7468-2

**Published:** 2019-06-28

**Authors:** Peng Jia, Fahui Wang, Imam M. Xierali

**Affiliations:** 1 Faculty of Geo-Information Science and Earth Observation (ITC), University of Twente, Enschede, 7500 The Netherlands; 2 International Initiative on Spatial Lifecourse Epidemiology (ISLE), Enschede, 7500 The Netherlands; 3 Department of Geography and Anthropology, Louisiana State University, Baton Rouge, LA 70803 USA; 4 Department of Family and Community Medicine, University of Texas Southwestern Medical Center, Dallas, TX 75390 USA

**Keywords:** Distance decay function, Hospital inpatient care, State Inpatient Database (SID), Hospital utilization, Florida

## Abstract

Understanding patients’ travel behavior for seeking hospital care is fundamental for understanding healthcare market and planning for resource allocation. However, few studies examined the issue comprehensively across populations by geographical, demographic, and health insurance characteristics. Based on the 2011 State Inpatient Database in Florida, this study modeled patients’ travel patterns for hospital inpatient care across geographic areas (by average affluence, urbanicity) and calendar seasons, and across subpopulations (by age, gender, race/ethnicity, and health insurance status). Overall, travel patterns for all subpopulations were best captured by the log-logistic function. Patients in more affluent areas and rural areas tended to travel longer for hospital inpatient care, so did the younger, whites, and privately insured. Longer travel distances may be a necessity for rural patients to cope with lack of accessibility for local hospital care, but for the other population groups, it may indicate rather better mobility and more healthcare choices. The results can be used in various healthcare analyses such as accessibility assessment, hospital service area delineation, and healthcare resource planning.

## Introduction

Spatial interaction models generally assume an inverse relationship between distance and interaction. Different population groups may exhibit distinctive travel patterns for healthcare visits, which can be analytically captured by distance decay functions. This study examines differential effects of distance decay on hospital inpatient visits among subpopulations. The differences may stem from their varying responses to physical distance to healthcare facilities such as hospitals, and reflect variable group mobility and possible scopes of healthcare choices.

Different travel patterns for hospitalization between the elderly and the overall population lead to different configuration of hospital markets. Jia et al. (2015) found that the commonly used *Dartmouth HSAs*, produced solely based on the Medicare hospitalization records (Center for Evaluative Clinical Sciences 1999), differed significantly from the hospital service areas (HSAs) based on the overall hospitalization records. Specifically, the number of Medicare-derived HSAs is higher (and thus a smaller area for each HSA on average) than the all-population-based HSAs since most of hospitalization of the elderly (aged ≥ 65) have occurred locally within a smaller spatial range (Jia 2016). Other studies suggest that spatial interactions between patients and hospitals are also affected by patients’ health insurance status, location of residence, and socio-demographic characteristics such as gender, race, and socioeconomic status (SES) (Basu and Cooper 2000; Biello et al. 2010; O’Neill 2004). Disparities in travel patterns can have an indirect and long-term impact on health outcomes for patients (O’Neill 2004). For example, compared to younger or overall patients, the healthcare travel behavior of elderly patients are more sensitive to increased distance to hospitals (Jia et al. 2017a). Moreover, travel behavior indicates how much patients rely on local hospitals for care and how vulnerable they are in responses to changes of local hospital market (e.g., reduced number of beds, hospital closures) (Escarce and Kapur 2009). Others focus on various factors affecting health-seeking behavior such as the study of outpatients in New York (Basu and Cooper 2000) and the research on inpatients in rural Pennsylvania and California (Escarce and Kapur 2009; O’Neill 2004).

In healthcare studies, at least three major issues need to be informed of the distance decay behavior of the population under investigation. Much of the debate on the best model for measuring spatial accessibility of healthcare services can only be settled by deriving “the best function to capture the distance decay behavior” in real-world healthcare utilization (Wang 2012). When using the Huff (1964) model for delineating HSAs, a critical task is to identify the spatial behavior of patients for hospital visits, which may not be best captured by a power function as assumed in the classic Huff model (Jia et al. 2017b). Furthermore, differing distance decay behaviors for general versus specialized care visits (i.e., a steeper-declining gradient for patients for general hospital care than those for more specialized care) lead to different average travel ranges, and thus form the theoretical foundation of a hierarchal central place structure in hospital systems (Jia et al. 2017a).

To our knowledge, few studies have examined the issue comprehensively across populations by geographical, demographic, and health insurance characteristics. The aim of this study is twofold: to identify the best-fitting distance decay functions for subpopulations and to examine how the distance decay behaviors differed among the subpopulations. The paper focuses on possible variability across geographic areas of average affluence and urbanicity levels, across calendar seasons, and across population subgroups in terms of age, gender, race/ethnicity, and health insurance status.

## Data sources and processing

Florida is situated in the southeastern US with three facets bordered by water: the Gulf of Mexico to the west, the Florida Straits between the USA and Cuba to the south, and the North Atlantic Ocean to the east. Hence, the edge effect regarding the tendency of patients to travel across state boundaries for hospital care is considered limited in Florida. This makes Florida an ideal study area for investigating patients’ healthcare travel patterns. According to the 2010 Census, Florida had a total population of roughly 18.8 million, with 57.9% whites, 16% blacks, 22.5% Hispanics, and 2.4% Asians.

Our primary data source was the State Inpatient Database (SID), as part of the Healthcare Cost and Utilization Project (HCUP) (Agency for Healthcare Research and Quality 2011). A total number of 2,376,743 inpatient discharge records from 22 acute long-term care hospitals and 199 general medical and surgical hospitals were extracted for Florida in 2011. Each record represents one inpatient discharge and includes a range of individual socio-demographic factors of the patient, such as age, gender, race/ethnicity, expected source of payment (e.g., health insurance type), ZIP code of residence (the finest level at which patients’ location of residence is available in SID), and hospital and diagnostic information for the visit, such as hospital identifier, primary and secondary diagnoses and procedures, admission and discharge date and status, length of stay, and total charges.

**Fig. 1:**
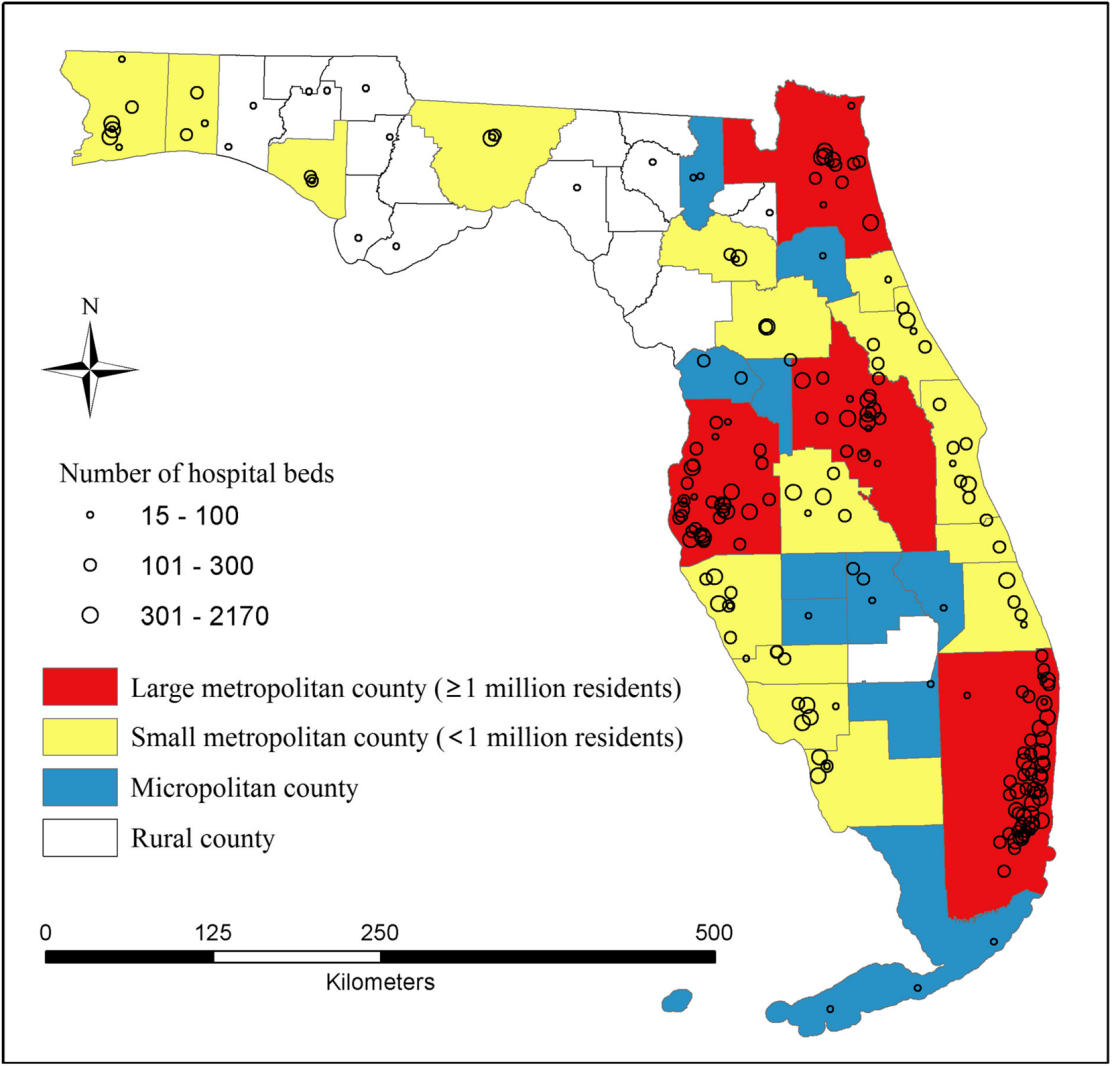
Hospitals across counties (by urbanicity) in Florida

We also used other datasets such as the 2010 US ZIP code boundaries, 2010 US census block boundaries with the number of total population and the population in each racial/ethnic category, and 2014 primary and secondary road networks (including maximum roadway speed) by the Florida Department of Transportation. The 2013 American Hospital Association’s (AHA) survey files were used to match with the SID file to define information on hospitals, such as hospital type and number of beds. Figure 1 uses different sizes of circles to represent hospital bed sizes.

There are three census units smaller than ZIP codes, though not completely nested within the boundaries of ZIP codes. In Florida, there were 4245 census tracts, 11,442 block groups, and 484,481 census blocks, but only 983 ZIP codes. Three data processing tasks were discussed elsewhere (Jia et al. 2018), but merit some discussion here. One was to estimate population at the ZIP code level (not directly available from the census). The *areal weighting interpolator* (Goodchild and Lam 1980) was used to interpolate population from census blocks (the smallest geographic units with census population counts) to ZIP codes. By overlaying the ZIP code and block layers, if a block was split among multiple ZIP code areas (termed *intersected zones*), its population was apportioned to each intersected zone based on its areal proportion over the block. The estimated population in all intersected zones within each ZIP code was then summed up to yield the population in that ZIP code. Another task was to generate the population-weighted centroids, instead of simply geographic centroids, to represent the locations of ZIP codes. This is particularly necessary in rural or peripheral suburban areas where population or business tend to concentrate in limited space (Luo and Wang 2003). Since census blocks are much smaller than ZIP codes (i.e., about 493 blocks in one ZIP code on average), it is acceptable to compute the population-weighted centroids of ZIP codes “based on block-level population data” (Wang 2015). The third issue was the computation of travel time between patients and hospitals. This study used the ArcGIS Network Analyst module to estimate the shortest path drive time between each pair of ZIP code centroids and hospitals.

In preparing for subsequent analysis of the gravity model in “Modeling hospital visits by gravity model,” patient hospital visits were aggregated by the unique pairs of ZIP codes (origins) and hospitals (destinations), i.e., O-D pairs. In other words, it was the volume of discharges from hospital *j* to ZIP code *i*, denoted by *T*_*ij*_. For the analysis of distance decay patterns by population subgroups in “Hospital utilization patterns by population subgroups,” patient hospital visits were first grouped by the subgroups and then cumulated inversely by travel time in minutes. For example, for all visits by male patients, the numbers of patient visits were obtained for traveling ≥ 60′, then ≥5 9′, ≥ 58′, and so on.

## Modeling hospital visits by gravity model

The analysis of distance decay in hospital inpatient visits begins with modeling the volumes of patient flows between patients and hospitals, and possible variability across geographic areas by average affluence levels and by urbanicity, and across calendar seasons.

### Gravity model and its estimation

The interaction between residents and hospitals, measured in the volume of discharges from hospital *j* to ZIP code *i*, denoted by *T*_*ij*_, is formulated as a gravity model:

1 $$ {T}_{ij}=\mu {P}_i^{\alpha }{S}_j^{\sigma }f\left({d}_{ij}\right) $$

where *P*_*i*_ is total population in ZIP code *i*, *S*_*j*_ is number of beds in hospital *j*, *α* and *σ* are parameters describing the effects of ZIP code population and hospital size upon the interaction, respectively, *μ* is a scalar, *d*_*ij*_ is travel time from ZIP code *i* to hospital *j* in minutes, and *f*(*d*_*ij*_) is a generalized distance decay function. In this study, *P*_*i*_, the population in ZIP code *i*, was interpolated from the block level, with its location represented by the population-weighted centroid, the address of hospital *j* was geocoded for its location, and travel time *d*_*ij*_ between them was estimated via the shortest path on the road network by assuming that travelers followed the posted speed limits.

The remaining question here is how to define the distance decay function *f*(*d*_*ij*_). In addition to the power function used in the classic Huff model, three other popular functions can be found in the literature. For example, Hodgson (1988) used a negative exponential function to explore the rural accessibility to healthcare in a developing country, Guagliardo (2004) used a Gaussian function to examine the accessibility of primary care providers within their practical service areas, and Delamater et al. (2013) used a log-logistic function to describe the distance decay of hospital utilization in Michigan. In summary, a total of four functions (power, exponential, Gaussian, and log-logistic) were tested in this study. As shown in Table 1, *β* was the only parameter in power and exponential functions, *θ* was the only parameter in Gaussian function, and log-logistic function had both parameters *β* and *θ*.

**Table 1:** Distance decay functions

Distance decay function	Formula *f*(*d*_*ij*_)	Parameter(s)
Power (P)	${d}_{ij}^{-\beta }$	*β*
Exponential (E)	${e}^{-\beta {d}_{ij}}$	*β*
Gaussian (G)	${e}^{-{\left({d}_{ij}/\theta \right)}^2/2}$	*θ*
Log-logistic (L)	1/(1 + (*d*_*ij*_/*θ*)^*β*^ )	*β*, *θ*

With *T*_*ij*_, *P*_*i*_, *S*_*j*_, and *d*_*ij*_ in Eq. (1) all assigned, we used the nonlinear least square (NLLS) regression to estimate the parameters *α*, *σ*, *β*, *θ*, and *μ* for each function. Note that the NLLS differs from the more commonly used ordinary least square (OLS) on a linearized form of the original nonlinear function. Take *f*(*d*_*ij*_) defined by a power function as an example, Eq. (1) could be transformed into a linear function by taking logarithms on both sides such as follows:

$$ \mathit{\ln}{T}_{ij}= ln\mu +\alpha ln{P}_i+\sigma ln{S}_j-\beta ln{d}_{ij} $$

It may be estimated by a regular OLS by minimizing the sum square error of *lnT*_*ij*_. It is different from the NLLS regression, which is to minimize the sum square error of *T*_*ij*_. The resulting estimate values for the parameters can be significantly different between NLLS and OLS, and their goodness-of-fit measures are also not comparable. This research used the NLLS regression to estimate the four distance decay functions, as we emphasized the fitness for the patient flow volume *T*_*ij*_ directly rather than its log-transform *lnT*_*ij*_. Furthermore, when *f*(*d*_*ij*_) takes the form of a Gaussian or log-logistic function, Eq. (1) cannot be linearized by log transformation and thus cannot be estimated by OLS. For comparability of the four functions, we also needed to use NLLS regression.

For OLS regression, a popular measure for a model’s goodness of fit is *R*^*2*^ (coefficient of determination), defined as the portion of dependent variable’s variation (termed “total sum squared (TSS)”) explained by a regression model (termed “explained sum squared (ESS)”), i.e., *R*^*2*^ *=* ESS/TSS *=* 1 − RSS/TSS. However, *R*^*2*^ is no longer applicable to nonlinear regression as the identity “ESS + RSS = TSS” no longer holds and the residuals do not add up to 0. Here, we used a *pseudo*-R^2^ (defined similarly as 1 − RSS/TSS) as “an approximate measure of goodness of fit” (Wang 2015:124). Another index for measuring the performance of a regression is Akaike information criterion (AIC), which measures the relative quality of the models due to varying complexities of four functions. In our case, there are four parameters in power, exponential and Gaussian models, and five parameters in log-logistic model. A smaller AIC indicates a simpler model, and is thus preferred. The model with the maximum *pseudo*-*R*^2^ and minimum AIC was selected as the best model. The NLLS regressions were implemented in *R* (Development Core Team 2011).

The *pseudo*-*R*^2^ and minimum AIC values for regressions of various functions are reported in Table 2. Note that the number of observations in each model was the number of non-zero patient flows between a ZIP code area and a hospital. For overall patients, the log-logistic function edged out the exponential function with the maximum *pseudo*-*R*^2^ and minimum AIC (top row in Table 1), and thus considered the best-fitting one. The estimated parameters in the log-logistic function are reported in Table 3.

**Table 2:** Fitness of the gravity model by geographic areas and calendar seasons

	No. obs. (*n*)	*pseudo*-R^2^				*Akaike Information Criterion* (AIC)			
		P	E	G	L	P	E	G	L
All ZIP code areas	37,216	0.29	0.50	0.47	0.50	509,491	496,514	498,070	496,504
Median household income									
0–25th	11,564	0.38	0.54	0.54	*0.55*	159,648	156,030	156,232	*155,974*
26th–50th	10,339	0.30	*0.50*	0.48	0.49	142,977	*139,524*	139,930	139,577
51st–75th	10,026	0.23	0.46	0.43	*0.46*	136,295	132,841	133,362	*132,783*
76th–100th	4780	0.41	*0.50*	0.47	0.49	59,582	*58,817*	59,051	58,859
Urbanicity									
Large metro	20,964	0.33	0.52	0.50	*0.52*	287,935	280,780	281,780	*280,779*
Small metro	11,913	0.27	*0.71*	0.70	0.70	163,582	*152,728*	153,078	152,853
Micropolitan	2894	0.63	0.82	0.83	*0.84*	35,644	33,577	33,478	*33,297*
Rural	1445	0.25	0.35	0.29	*0.37*	17,212	17,020	17,137	*16,982*
Calendar season									
Jan–Mar	20,402	0.29	0.47	0.45	*0.47*	235,335	229,309	230,222	*229,263*
Apr–Jun	20,732	0.29	0.48	0.45	*0.48*	232,770	226,553	227,474	*226,489*
Jul–Sep	20,652	0.30	0.48	0.46	*0.48*	235,933	229,627	230,575	*229,554*
Oct–Dec	20,608	0.29	0.47	0.45	*0.47*	236,250	230,158	231,048	*230,103*

P, E, G, and L for power, exponential, Gaussian, and log-logistic distance decay function, respectively; the best-fitting model (max *pseudo*-*R*^2^ and min AIC) in italics

**Table 3:** Average travel time, and parameters in the log-logistic function in the gravity model by geographic areas and calendar seasons

	No. patients	T_all^a^	T_60^b^	*μ*	*α*	*σ*	*θ*	*β*
All ZIP code areas	2,376,743	17.6	13.3	0.20	0.66	0.40	6.29	2.14
Median household income								
0–25th	827,281	16.4	11.4	0.59	0.57	0.36	6.56	2.52
26th–50th	718,694	17.7	13.7	1.00	0.48	0.46	6.53	2.14
51st–75th	617,952	18.3	14.5	0.06	0.77	0.40	6.12	2.02
76th–100th	207,852	20.0	15.2	4.3e-5	1.37	5.26	7.59	2.22
Urbanicity								
Large city	1,497,608	13.9	12.1	0.01	0.90	0.45	5.97	2.22
Small city	719,150	20.5	14.3	0.02	0.85	0.47	12.06	2.55
Micropolitan	109,831	34.2	20.2	3.7e-3	1.12	0.40	14.53	3.20
Rural	50,154	50.9	28.0	0.02	0.83	0.73	11.20	1.82
Calendar season								
Jan–Mar	608,851	17.5	13.3	0.10	0.61	0.38	5.96	2.01
Apr–Jun	583,737	17.7	13.3	0.08	0.63	0.39	6.01	2.01
Jul–Sep	588,641	17.7	13.3	0.06	0.65	0.40	6.08	2.02
Oct–Dec	595,514	17.7	13.3	0.07	0.64	0.39	6.15	2.03

^a^T_all, average travel time in minutes for all patients; ^b^T_60, average travel time in minutes for patients traveling ≤ 60 min

### Variability by geographic areas and by calendar season

To examine the variability of the distance decay effect, we further estimated the gravity model in Eq. (1) on various groupings based on patients’ ZIP code areas. First, all 983 ZIP codes were classified into four groups by a national quartile classification of the ZIP code median household income, as a proxy for measuring average socioeconomic status (SES) in neighborhoods. The quartiles are defined as 1 (< $39,000/year), 2 ($39,000—47,999), 3 ($48,000—62,999), and 4 (≥ $63,000). Secondly, the ZIP code areas were grouped into four urbanicity categories: large metropolitan (≥ 1 million residents), small metropolitan (50,000—1 million residents), micropolitan (10,000—49,999 residents), or rural areas. Figure 1 shows four different urbanicity levels at the county level in Florida. Thirdly, all records were divided into four calendar seasons in which each hospital visit occurred (January–March, April–June, July–September, and October–December).

Based on the regression results on the fitness of various functions reported in Table 2, the log-logistic model had the highest *pseudo*-*R*^2^ and lowest AIC with a slight edge over the exponential model among most subpopulations, except those in small metropolitan and the second and fourth SES quartiles where the exponential function performed slightly better. The power function traditionally adopted in a gravity model produced the lowest *pseudo*-*R*^2^ and the highest AIC. For consistency and comparison across all subpopulations, we chose the log-logistic model as the best-fitting model for subsequent discussions. The regression results including estimated parameter values for the log-logistic model are reported in Table 3.

**Fig. 2:**
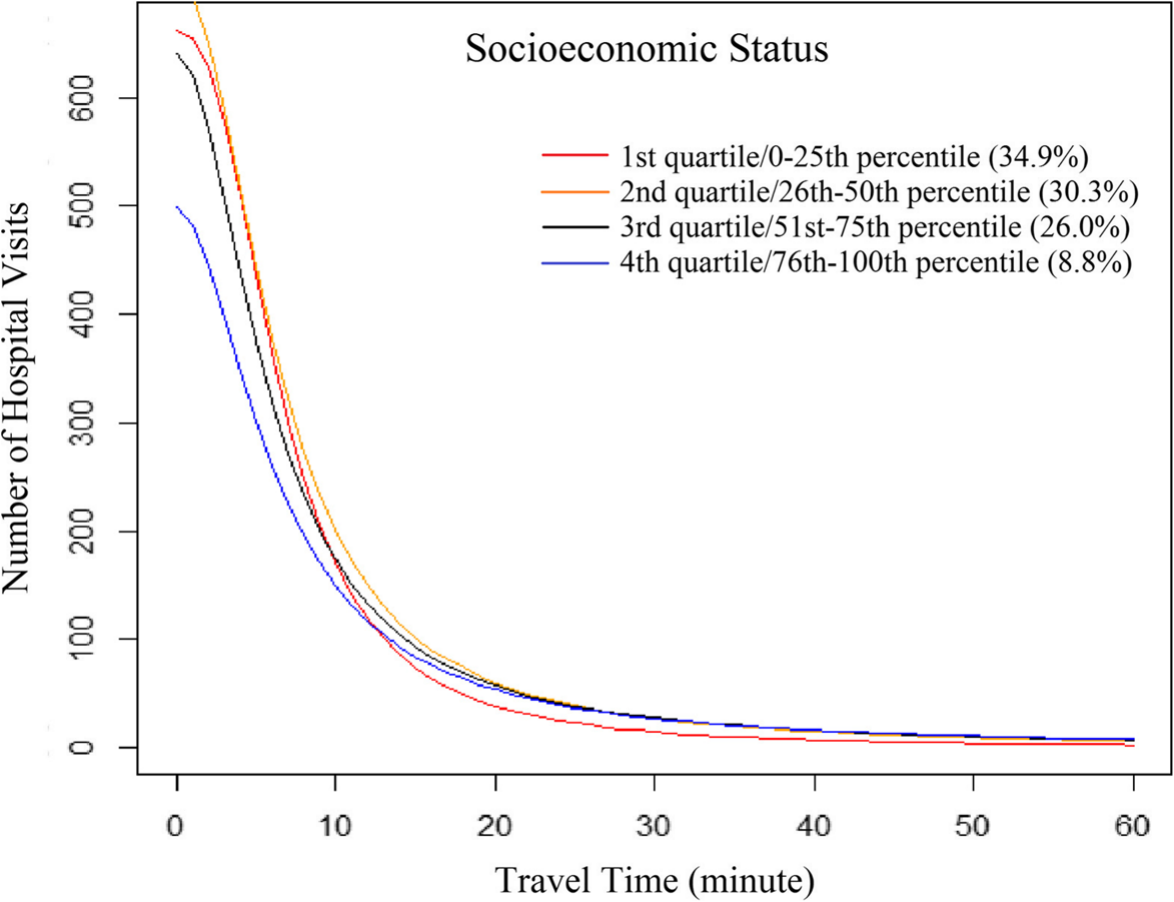
Travel patterns of patients across ZIP code SES subgroups, fitted by the log-logistic function

**Fig. 3:**
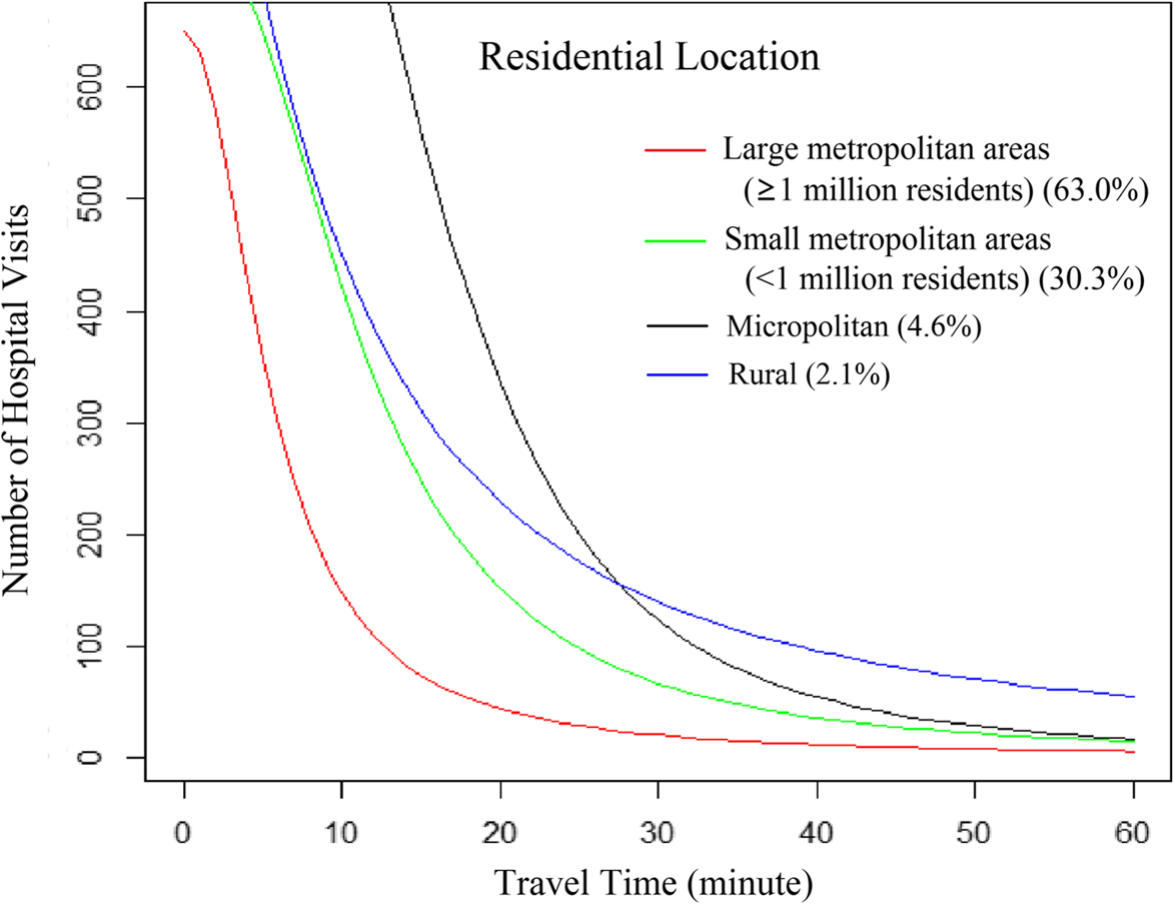
Travel patterns of patients across ZIP code urbanicity groups, fitted by the log-logistic function

Figures 2 and 3 were designed to illustrate various travel patterns captured by the fitted log-logistic functions. The optimal log-logistic curve for each subgroup was drawn with *P*_*i*_ and *S*_*j*_ set as 10,000 and 100, respectively, for highlighting the effects of increasing travel time on decreasing hospital visits. In general, an increase in *α* or/and *σ*, as an exponent of the number of population within ZIP codes and of the number of hospital beds respectively, leads to a larger number of hospital visits from a ZIP code to a hospital. An increase in *θ* similarly results in more hospital visits. However, as *β* increases, the distance decay effect becomes stronger with a more rapid decline in the number of hospital visits with travel time. Given the same *α*, *σ*, and *θ*, an increase in *β* leads more patients to travel shorter and fewer patients to travel longer for hospital visits. The synergetic effects caused by respective changes in these four parameters are more complex, which can be observed through comparison of the fitted curves.

In addition, average travel time for patients in each subgroup was calculated and reported in Table 3. For example, the average travel time was 17.6′ for all patients, and 13.3′ for patients traveling 60 min or less. We chose 60 min as an importance benchmark since most patients traveled no more than 60 min for hospital visits (Delamater et al. 2013). The average travel time gives us some intuitive understanding of the travel burden for the group relative to other groups. However, one needs to consult the analytic functions and their visualized patterns in Figs. 2 and 3 to have a complete picture of the whole spectrum of distribution across various travel ranges.

For ZIP code areas, average travel time increased from the first to fourth income levels for both overall patients (from 16.4 to 20.0 min) and those only traveling 60 min or less (from 11.4 to 15.2 min). Also, the distance decay factor *β* decreased from the first to third bracket and bounded up in the fourth (Table 3), implying a gradually decreasing distance decay effect before reaching the most affluent neighborhoods. As shown in Fig. 2, a larger number of hospital visits occurred within 15 min from patients’ residence in the first quartile, and much fewer of them occurred beyond 15 min relative to other quartiles.

There were 190 out of 221 hospitals (86%) located in metropolitan areas, with 123 out of the 190 hospitals (65%) in areas with a population of over 1 million. For the overall patients, the average travel time gradually increased with residential location becoming less urbanized, from large metropolitan (13.9 min), small metropolitan (20.5 min), and micropolitan (34.2 min), to rural areas (50.9 min) (Table 3). A consistent trend was also observed among patients traveling 60 min or less, from 12.1 min in large metropolitan areas to 28.0 min in rural areas. As shown in Fig. 3, hospital visits by patients in different subgroups tended to be concentrated at different locations along the axis of travel time. Most hospitalization in large metropolitan areas occurred within 10 min from patients’ residence, especially within 5 min where few hospital visits occurred for patients in other subgroups. Most patients in small metropolitan areas spent 5–20 min traveling to hospitals, while most micropolitan patients spend 15–30 min on traveling. Although hospitalization of some rural patients occurred at a closer distance from their residence relative to micropolitan patients, an apparently larger proportion of rural patients spent ≥ 30 min than any other urban subgroup. In terms of distance decay effect, the number of hospital visits declined most rapidly with time in micropolitan areas (*β* = 3.20), followed by small (*β* = 2.55) and then large metropolitan areas (*β* = 2.22). Rural patients showed the weakest distance decay effect (*β* = 1.82), but also with the lowest fitting power (*pseudo*-*R*^2^ = 0.37).

There were no significant differences found in travel patterns among patients discharged in four calendar seasons. The distance decay factor was fairly stable, ranging from 2.01 during January–March to 2.03 during October–December (Table 3).

## Hospital utilization patterns by population subgroups

### Cumulative probability approach

The numbers of subpopulations with different ages, genders, races/ethnicities, and health insurance coverage are not available at the census block level, and thus cannot be transferred to ZIP codes. Therefore, it is not feasible to model the patient-hospital interaction based on the gravity model in Eq. (1). Here, we adopt the cumulative probability approach (Delamater et al. 2013), equivalent to a reversed cumulative distribution function, for describing the decay effects of hospital utilization with travel time in those subpopulations. The distance decay function is correspondingly re-formulated as follows:

2 $$ {Y}_d=\mu f\ (d) $$

where *d* is travel time from hospitals to patients’ home ZIP codes in minutes, *Y*_*d*_ is the total proportion of discharges from hospitals that are more than *d* minutes’ drive from their home ZIP codes, where *Y*_*d*_ approaches 1 as *d* approaches 0 while approaching 0 as *d* approaches the longest travel time by the patients included; *f*(*d*) represents a generalized distance decay function, with four candidates in this study (Table 1); and *μ* is a scalar parameter to be estimated together with other parameters in *f*(*d*).

**Fig. 4:**
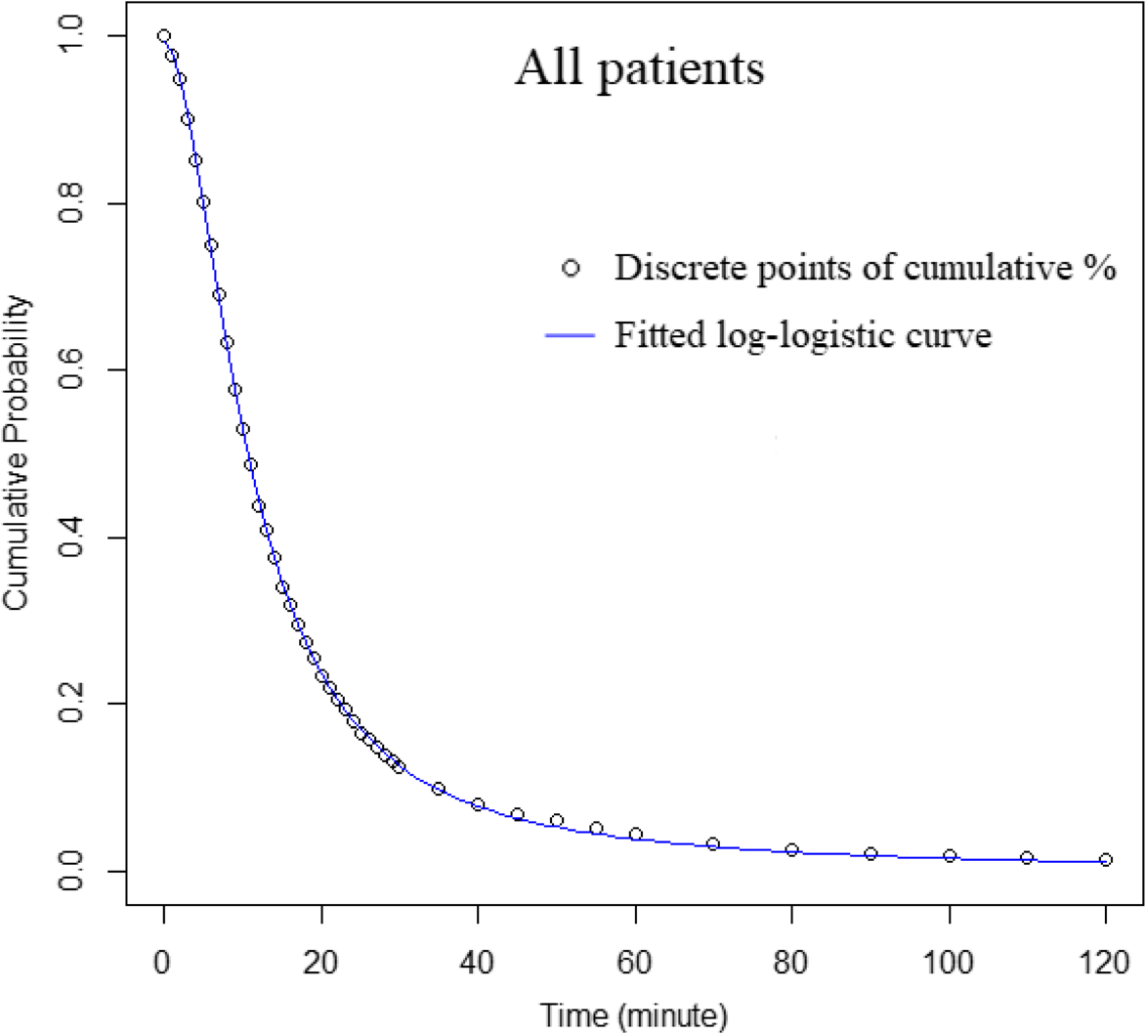
Hospital utilization patterns of all patients, fitted by the log-logistic function

As explained in “Data sources and processing,” patient hospital visits were aggregated inversely by travel time in minutes, and corresponding cumulative ratios (probabilities) were calculated. For example, as shown in Fig. 4, the cumulative probabilities for all patients declined with travel time in minutes. The data points were defined for every minute for the range [0′, 30′] (31 points), every 5 min from (30′, 60′] (6 points), and then every 10 min for (60′, 120′] (6 points). As travel time increased, the number/proportion of patients traveling beyond that time became smaller, and thus points were set farther apart with longer intervals. Therefore, the number of observations (*n*) was 31 + 6 + 6 = 43 for the overall model and each subgroup (Table 4). Similarly, the NLLS regression was used to estimate the parameters, and *pseudo*-*R*^2^ and AIC were calculated to identify the best-fitting models. Based on the regression results reported in Table 4, the log-logistic function produced the best fit with a *pseudo*-*R*^2^ as high as 0.9999. A good fit for regression by the cumulative probability approach is common since the values on the *y*-axis are cumulative and monotonically decline. A similar study (Delamater et al. 2013) also reported “an excellent curve fit” by the same function. The near-perfect fitting by the log-logistic function across all subpopulations in this study was impressive, and demonstrated its advantage over other functions (Table 4).

**Table 4:** Fitness of four distance decay functions for hospital utilization of subpopulations by age, gender, and health insurance coverage

	No. obs. (*n*)	*pseudo*-*R*^2^				Akaike information criterion (AIC)			
		P	E	G	L	P	E	G	L
Overall	43	0.1559	0.9928	0.9575	*0.9999*	17.546	187.510	110.930	*353.748*
Age									
< 12	43	0.1283	0.9889	0.9749	*0.9997*	23.632	164.052	128.915	*320.540*
< 12^*^	43	0.1213	0.9909	0.9544	*0.9994*	20.355	176.046	106.907	*291.979*
12–17	43	0.1199	0.9894	0.9610	*0.9993*	21.699	168.408	112.301	*285.406*
18–24	43	0.1451	0.9911	0.9618	*0.9997*	19.668	176.536	113.940	*327.926*
25–34	43	0.1411	0.9910	0.9706	*0.9998*	21.282	174.571	123.882	*335.221*
35–44	43	0.1500	0.9925	0.9607	*0.9999*	18.693	184.881	113.495	*354.470*
45–54	43	0.1597	0.9927	0.9488	*0.9998*	16.159	187.992	104.167	*344.917*
55–64	43	0.1582	0.9923	0.9489	*0.9998*	16.364	185.307	104.104	*332.716*
65–74	43	0.1583	0.9926	0.9518	*0.9998*	16.546	186.944	106.412	*333.815*
≥ 75	43	0.1770	0.9928	0.9582	*0.9997*	14.633	188.917	113.548	*319.110*
Gender									
Male	43	0.1550	0.9907	0.9361	*0.9997*	7.567	312.621	175.721	*565.894*
Female	43	0.1550	0.9927	0.9610	*0.9999*	18.157	185.929	114.076	*356.452*
Race/ethnicity									
White	43	0.1504	0.9932	0.9539	*0.9998*	17.552	190.456	107.715	*350.319*
Black	43	0.1664	0.9900	0.9652	*0.9997*	17.717	172.448	118.810	*322.553*
Hispanic	43	0.1670	0.9923	0.9732	*0.9990*	17.723	183.753	130.126	*268.489*
Asian	43	0.1409	0.9856	0.9829	*0.9992*	23.354	152.463	144.953	*276.025*
Native	43	0.1458	0.9835	0.9296	*0.9965*	15.640	154.031	− 91.664	*218.778*
Others	43	0.1347	0.9890	0.9681	*0.9993*	21.059	166.722	120.898	*282.416*
Health insurance									
Medicare	43	0.1688	0.	0.9535	*0.9997*	15.433	187.778	108.584	*328.147*
Medicaid	43	0.1502	0.9926	0.9605	*0.9999*	18.965	185.123	113.038	*356.729*
Private	43	0.1336	0.9901	0.9695	*0.9997*	21.713	170.622	122.122	*326.438*
Self-pay	43	0.1651	0.9930	0.9494	*0.9998*	15.413	190.383	105.171	*347.304*
No charge	43	0.1774	0.9911	0.9745	*0.9992*	16.346	178.100	133.068	*278.631*

Numbers in italics indicate the best fitting model in pseudo-R2 and AIC

P, power function; E, exponential function; G, Gaussian; L, log-logistic function

^*^Newborn hospital visits were excluded

The optimal log-logistic curve for each subpopulation was also drawn for highlighting the effects of increasing travel time on decreasing hospital visits. Each parameter independently influenced the shape of the curve in a different way. As *β* increased, the decay effect became more intensive with more hospitalization occurring close to the residence of patients. A standalone increase in *μ* or *θ*, with other parameters remaining constant, both corresponded to a larger proportion of hospitalization occurring away from patients’ residence. Unlike *T*_*ij*_ with a relatively unlimited upper bound, *Y*_*d*_ represented a proportion over a fixed number of patients, thus the changes occurring on the end of short travel time also affected the proportion on the end of long travel time. This resulted in more complex synergetic effects on the shape of curves by the change of each parameter, let alone the changes in all parameters, which, however, were subsequently scrutinized in patterns among subpopulations by health insurance coverage.

### Variability by subpopulations

**Fig. 5:**
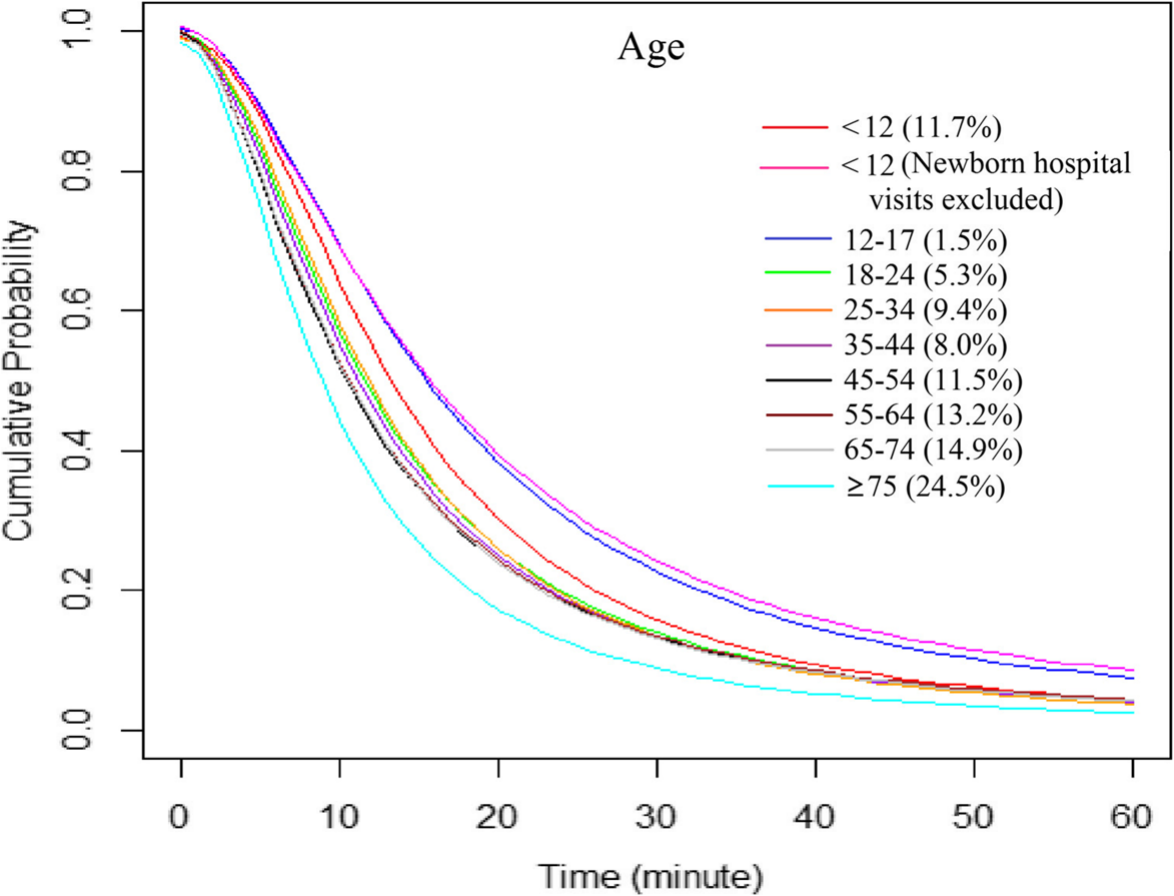
Hospital utilization patterns of patients across age groups, fitted by the log-logistic function

**Fig. 6:**
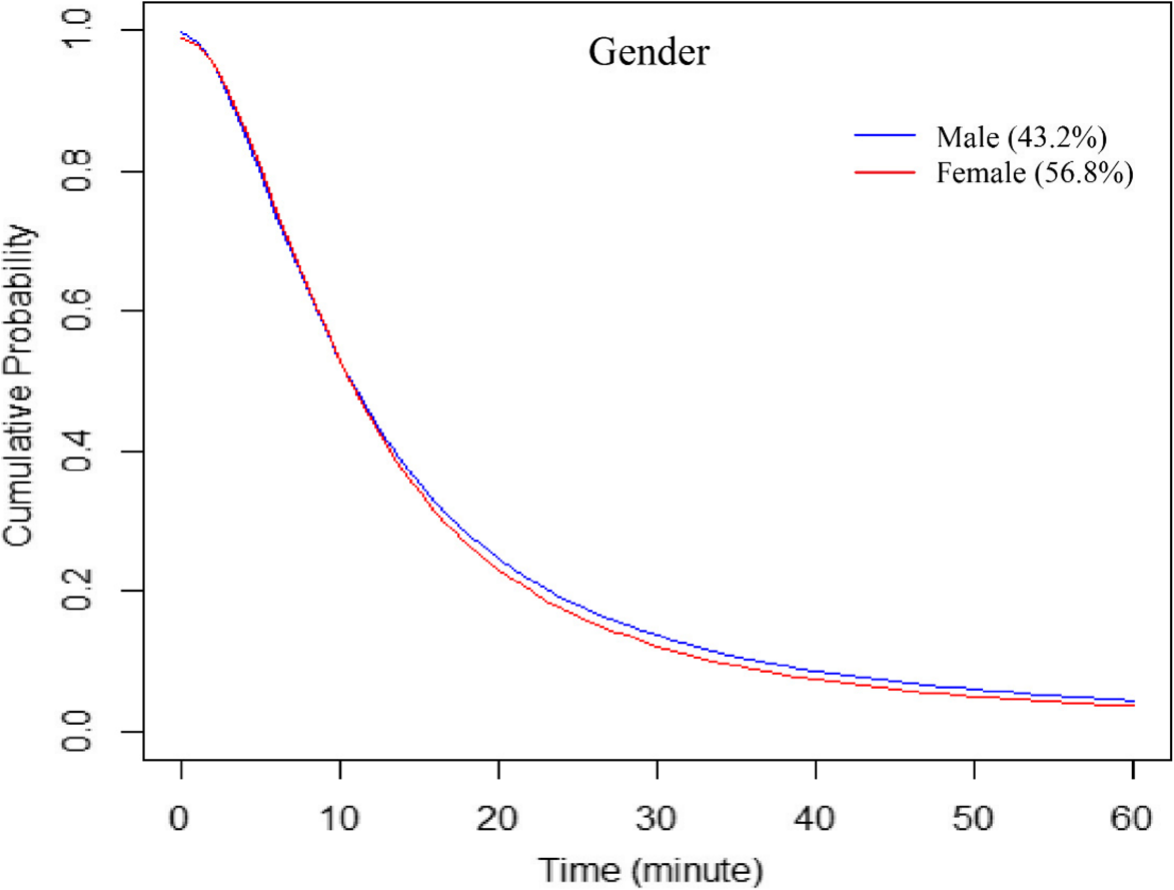
Hospital utilization patterns of patients across gender groups, fitted by the log-logistic function

A general trend of increasing distance decay effects with increasing age of patients was observed, with the elderly (age ≥ 75) most affected and the younger group (age < 18) least affected by the increased travel time to hospitals (Fig. 5). The distance decay curve representing those younger than 12 declined less sharply after excluding birth visits. No apparent differences were observed between genders, although females were slightly less likely to travel longer than males (Fig. 6).

**Fig. 7:**
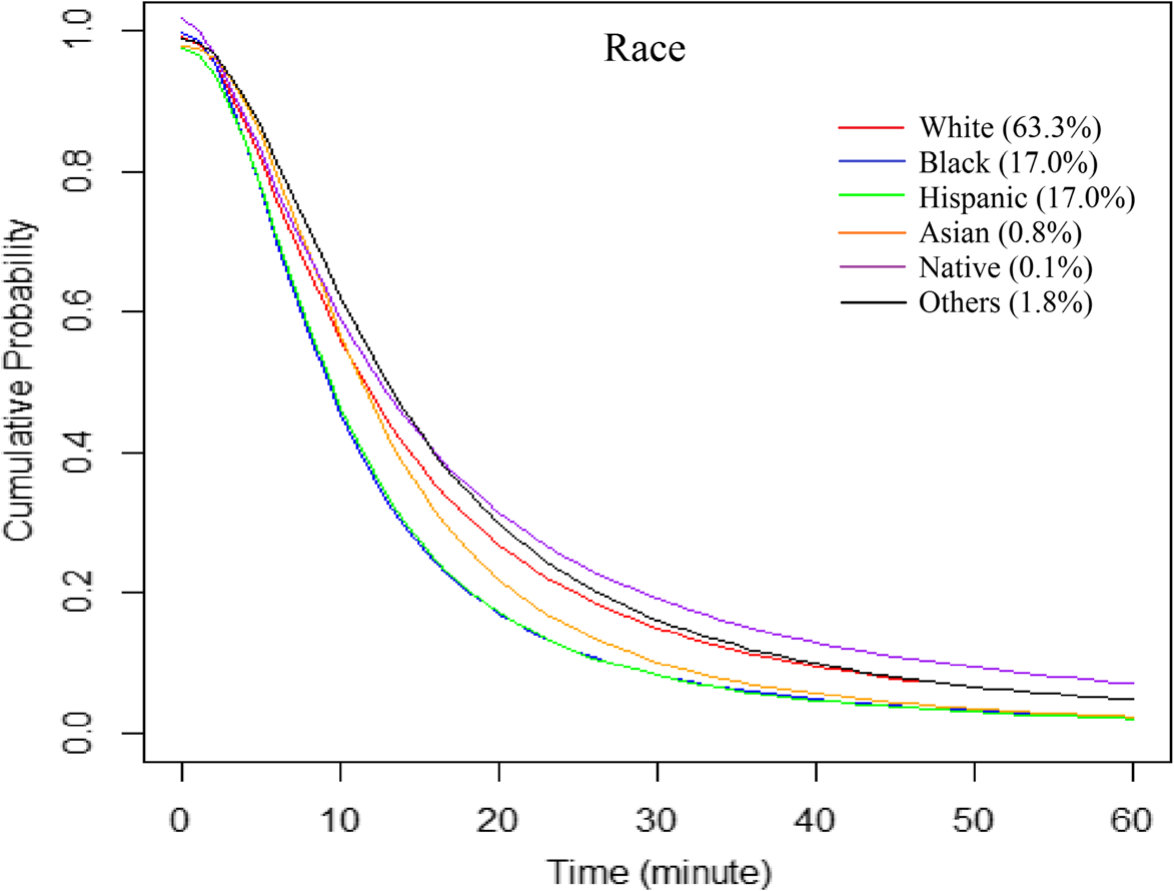
Hospital utilization patterns of patients across racial/ethnic groups, fitted by the log-logistic function

Blacks and Hispanics had a similar hospital utilization trend which declined fastest, and the hospital utilization decreased faster with travel time among Asians than whites (Fig. 7). Native Americans included in this study traveled the longest time on average (23.9 min) for hospitalization services. Whites spent the second longest travel time to hospitals on average (19.2 min), followed by Asians (16.7 min), blacks (14.7 min), and Hispanics (14 min). A considerable number of whites traveled more than 30 min to get to hospitals, while the numbers of patients in other racial/ethnic groups started to decline to a similar level when the required travel time increased to about 30 min (Fig. 7). For patients traveling 60 min or less, whites (14.2 min) and Asians (13.4 min) on average consistently traveled the longest, but Hispanics (11.6 min) conversely traveled longer than blacks (11.5 min) on average, which corresponded to the order of distance decay effects. Due to relatively small numbers of discharge records from Asians and Native Americans, especially an extremely low *pseudo*-*R*^2^ for Native Americans, results for those two subgroups may not be representative of their entire racial groups, and hence needed to be treated with caution.

**Fig. 8:**
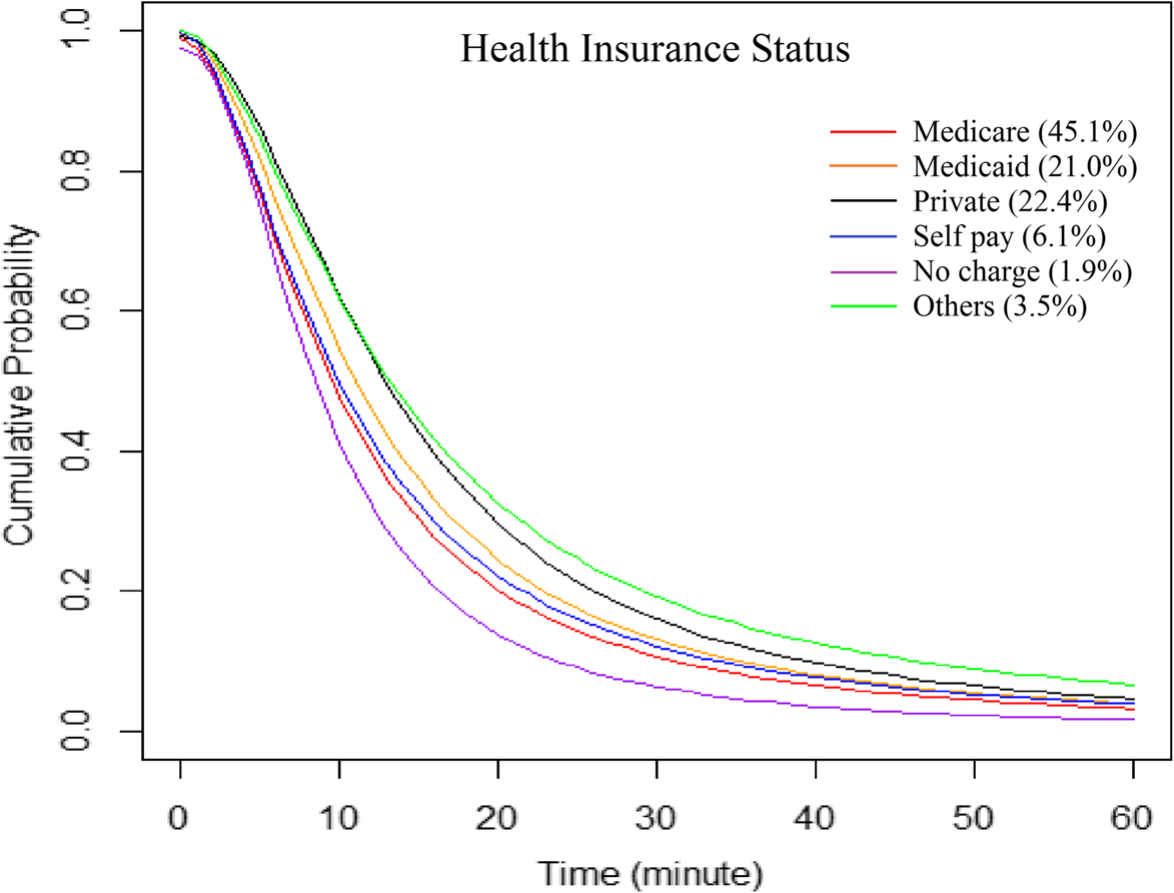
Hospital utilization patterns of patients across insurance (payment) groups, fitted by the log-logistic function

Patients paid by different health insurance plans for hospital services demonstrated diverse patterns (Table 5). The no-charge patients, those discharged without paying hospital bills, spent the shortest time to hospitals on average (12.6 min), followed by Medicare beneficiaries (16.0), self-pay patients (17.5), Medicaid beneficiaries (17.6), and privately insured (20.8). A consistent order among subpopulations was also observed in average travel time of those traveling 60 min or less (Table 5) and from the optimal curves for patterns of hospital utilization (Fig. 8).

**Table 5:** Average travel time, and parameters and fitness of the log-logistic function for hospital utilization patterns of subpopulations by age, gender, race/ethnicity, and health insurance coverage

	No. patients	T_all^a^	T_60^b^	*μ*	*θ*	*β*
Overall	2,376,743	17.6	13.3	0.992	10.725	1.870
Age						
< 12	278,094	19.8	15.5	0.992	13.332	2.056
< 12 (no newborn)^*^	75,696	26.3	17.4	1.005	15.566	1.769
12–17	35,232	25.3	17.3	1.003	15.387	1.852
18–24	126,525	19.4	14.0	0.997	11.573	1.912
25–34	222,157	18.2	14.2	0.989	11.912	2.011
35–44	190,860	18.5	13.7	0.993	11.211	1.892
45–54	273,250	18.2	13.2	0.999	10.437	1.788
55–64	313,738	18.6	13.3	1.000	10.577	1.789
65–74	353,623	18.1	13.3	0.995	10.602	1.817
≥ 75	583,227	14.3	11.5	0.983	8.993	1.933
Gender						
Male	1,026,589	18.3	13.4	0.998	10.695	1.790
Female	1,350,140	17.1	13.2	0.990	10.710	1.914
Race/ethnicity						
White	1,489,589	19.2	14.2	0.993	11.528	1.809
Black	400,428	14.7	11.5	0.997	9.152	2.027
Hispanic	399,395	14.0	11.6	0.974	9.540	2.089
Asian	19,837	16.7	13.4	0.977	11.526	2.263
Native	3343	23.9	14.2	1.017	12.208	1.631
Others	41,976	21.3	14.9	0.990	13.032	1.964
Health insurance						
Medicare	1,074,328	16.0	12.3	0.990	9.632	1.867
Medicaid	498,513	17.6	13.6	0.996	11.055	1.896
Private	531,698	20.8	15.2	0.991	12.987	1.977
Self-pay	144,432	17.5	12.6	0.998	9.951	1.797
No charge	45,300	12.6	10.7	0.974	8.641	2.146

^a^T_all, average travel time in minutes for all patients; ^b^T_60, average travel time in minutes for patients traveling ≤ 60 min

## Discussion

Patients’ travel behavior for seeking healthcare services underlies the healthcare market. More importantly, understanding differences in travel behavior among diverse subpopulations is vital for equitable health resource allocation. This study examined patients’ travel patterns of hospital utilization for diverse subpopulations, and compared them under each primary category (age, gender, race/ethnicity, SES, health insurance status, urbanicity, and calendar season). The log-logistic function was found to best capture the distance decay effects in patterns of patients’ travel for hospital visits in nearly all subpopulations, which is consistent with previous findings for the overall population (Delamater et al. 2013; Jia et al. 2017a, b).

The comparison among racial/ethnic subgroups reveals that mobility for seeking hospital services is more limited for blacks and Hispanics than for whites and Asians. Compared with a previous study focusing on the healthcare travel patterns of only congestive heart failure patients (Jia and Xierali 2015), this study found a similar pattern among racial/ethnic groups. The longest average travel time by Native Americans should be interpreted with caution due to a smaller sample size of this group. The fact that most Native Americans live in rural areas may provide additional explanations for a longer average travel time to hospitals (Lester 1999). Further research using datasets with larger numbers of Native Americans (also more Asians) is needed to better reveal their travel patterns for hospital visits.

There are at least three reasons that could help explain that rural patients may disproportionately travel longer for hospitalization services. First, rural residents are generally farther from their nearest hospitals than others. Secondly, while there are not many rural hospitals, most are small (< 100 beds) and provide limited services. Rural patients on average are much older, poorer and sicker, and have more complex health service needs that are only available in larger hospitals in major cities. Thirdly, rural residents are more likely to be uninsured than their urban counterparts (24% versus 18%) (Foundation 2003), and hence might bypass the nearest hospitals to seek care in other non-for-profit hospitals for lower charges (Jackson et al. 2002). Roughly 20% of the US population live in rural areas (Escarce and Kapur 2009), and their rural hospital bypass behavior has been of great interest to some researchers. In Colorado, about 45% of rural patients bypassed their local hospitals during the 1990s (Roh and Lee 2006). According to the inpatient discharge data from seven states, bypass rates among rural patients in 1991 (1996) are 29% (32%) in California, 35% (36%) in Florida, 25% (27%) in Maine, 25% (25%) in New York, 32% (30%) in Oregon, 32% (30%) in South Carolina, and 34% (34%) in Washington (Radcliff et al. 2003). In 2000, two thirds of rural hospitalization records were discharged from urban hospitals in California (Escarce and Kapur 2009). A study in England revealed that rural patients traveled longer than urban patients, where the 75th percentile in the distribution of distance traveled by rural patients was equivalent to the 90th percentile by urban patients (Propper et al. 2007). Rural patients normally perceive urban providers to be more qualified for delivering complex surgical services (Radcliff et al. 2003). These previous studies may also support the finding of this study. This creates a major challenge for rural hospitals as they may suffer from a heavy loss of patients and revenue, have to cut back on services, and then are under pressure to close.

Patients from wealthier neighborhoods were found more likely to travel longer than those from poorer neighborhoods. On one side, the wealthier neighborhoods are usually at a farther distance away from hospitals than are inner-city poor neighborhoods. A plethora of wealthy neighborhoods do not even have any hospital within their own ZIP codes, thus have to bear with this longer distance. On the other side, patients from neighborhoods of higher average socioeconomic status tend to have better mobility and can afford traveling longer for better services, instead of being limited to services provided by local hospitals only. A study of inpatients in England consistently revealed that the poor patients still traveled shorter than their affluent counterparts, after controlling for distance to hospital (Propper et al. 2007). In addition to a direct effect on the affordability of high costs caused by long travel (e.g., car ownership, patients’ travel cost, and family members’ visit costs), SES could also underlie or associate with other factors such as race/ethnicity, health insurance status, and location of residence to affect the travel behavior of the patients indirectly and health inequalities ultimately (Link and Phelan 1995).

Our results about travel behavior across health insurance coverage reinforce the previous findings that, patients covered by private insurance or managed care plans were more likely to bypass local hospitals and travel longer for hospitalization than Medicare and Medicaid beneficiaries and the uninsured (e.g., self-pay and no-charge patients) (Escarce and Kapur 2009; Radcliff et al. 2003). There are two types of no-charge patients, charity care and bad debt. Hospitals normally know at the time of admission that it is unlikely to be paid for the former, but do not know for the latter (Jackson et al. 2002). The main reason of failure to make payment for no-charge patients is lack of financial means or health insurance. The proportion of uninsured populations has been negatively associated with the level of household income (Jackson et al. 2002), which reflects the underlying impacts of SES on health insurance and hospital payment.

Black and Hispanics are significantly more likely than whites to be uninsured, so they are more likely to end up with no charge at the time of discharge, which explains the strongest distance decay effect of the no-charge subgroup from a racial, or more basically, socioeconomic aspect. Despite chance of lack of health insurance, self-pay patients may have a better ability to travel than the no-charge uninsured patients. The possibility cannot be ruled out that, in some cases, patients may withhold their health insurance status from hospital for getting the discounted prices (i.e. cash price), which could be significantly lower than the contract rate if paying coinsurance. Age, severity of illness, and bill charge may also confound the results; a comparison of hospital utilization patterns between no-charge and self-pay patients needs to be examined in future studies. Additionally, the interplay between the insured and uninsured may play a role in diverse travel patterns: no-charge patients may prefer to go to local hospitals they are more familiar with for saving the costs of traveling; hence, hospitals may charge the insured more than expected to cover the costs of the uninsured and balance their revenue (Buntin 2014), which could turn more insured patients away from local hospitals. Yet, this needs more longitudinal studies on hospital charges to confirm. Some other intra-insurance trends are not examined in this study due to lack of relevant information, such as shorter distances traveled by Medicare HMO (health maintenance organization) enrollees than Medicare FFS (fee-for-service) inpatients (O’Neill 2004).

It is worth noting that Florida is a special case in terms of population composition within the USA, where a number of seasonal residents originally from other states only spend their winter in Florida, especially the elder population that are more vulnerable to many health risks than other groups. The SID data do not identify whether a patient is a seasonal or permanent resident in Florida, so it is not feasible to directly examine possible differences in travel behavior between seasonal and permanent residents. However, SID data contain a time stamp marking the calendar season in which each hospital visit occurred (January–March, April–June, July–September, or October–December), which enables us to examine variation in travel patterns among four calendar seasons. Our results reported a stable travel pattern across four calendar seasons, which strictly speaking, however, cannot infer any differences between permanent and seasonal residents in their travel behavior in seeking hospital care. Also, this seasonal stability in travel behavior may not be generalizable to other states with a harsh winter climate without caution.

Here, a couple of caveats merit clarification. The population numbers within ZIP codes were estimated without detailed information of population distribution, which could be improved by using new methods and additional ancillary data (Jia et al. 2014; Jia and Gaughan 2016; Krivoruchko et al. 2011). Travel time was estimated as driving time on roadways with posted speed limits. A previous study by Wang and Xu (2011) suggested that *ArcGIS* (ESRI: Redland CA) tended to underestimate the travel time which drivers actually experienced when road conditions were considered (e.g., by Google Maps), but discrepancies were largely consistent with a gap close to 5 min. Therefore, the estimated travel time was appropriate for planning purposes as our study focused on the difference in travel impedance across ZIP codes. The main limitation is rather the lack of consideration of other travel modes such as public transits, upon which various disadvantaged groups may disproportionately rely. Also, the SID did not contain patient address information and precluded us from such an analysis. Furthermore, this study classified the 983 ZIP codes into four SES subgroups based on a national (instead of Florida’s) quartile classification of the median household income, available in the SID. However, such a strategy was considered acceptable given a similar median household income in Florida ($47,507) as the national one ($47,999), and similar counts of ZIP codes in four quartiles yielded by this classification.

## Concluding remarks

This study provides a comprehensive examination of travel behaviors of hospital inpatient visits across demographic, socioeconomic, geographic, and health insurance subgroups of patients. A major finding is that the log-logistic function better fits the patterns of patients’ travel and hospital utilization in almost all subgroups. A continuous distance decay function is an analytic and more accurate characterization of the complexity of patients’ travel behaviors than a simple measure of their mean travel time. Five key trends among subpopulations are observed:

1. Distance decay effects generally increased as the age of the patients increased.
2. Whites spent the longest travel time to hospitals on average, followed by Asians, blacks, and Hispanics.
3. Patients’ average travel time increased and distance decay effects decreased as their SES improved.
4. Patients spent longer travel time to hospitals on average as the urbanicity level of their residence decreased from large metropolitan to rural areas.
5. No-charge patients spent the shortest travel time to hospitals on average, followed by Medicare beneficiaries, self-pay patients, Medicaid beneficiaries, and the privately insured.

The results and methods used in this study can (1) help researchers choose the best-fitting distance decay function in modeling patients’ healthcare-seeking behavior in accessibility study, modeling patient flows, and defining hospital service areas; (2) identify distinctive “activity space” for hospital inpatient visits by various subpopulations, which to some extent reflects their corresponding mobility capacity and scope of healthcare choices; and (3) help to assess possible impacts of various hospital planning scenarios on specific target population groups, such as hospital closure, scaling-down or expansion of existing hospitals, and opening of new hospitals.

Future studies seem warranted to examine the following points. First, it remains unclear how different demographic, socioeconomic, and geographical determinants interplay with each other and influence patients’ travel patterns simultaneously. One way is to examine in depth the intra-variability of travel patterns in each population subgroup. For example, the poor rural patient may travel short distances to local hospitals, if any, but the affluent rural patient may bypass the local ones. Another approach is to employ a multivariate regression model to examine the collective effects of various attributes of patients. Secondly, the degree of dependency on local hospitals needs to be quantified for different population subgroups. Given that some disadvantaged populations coincide socioeconomically or geographically, their synergic effects on healthcare travel patterns could be amplified. For example, the poor rural people may undergo double stress from limited economic capacities and geographical isolation, which requires them to overcome more barriers than their inner-city counterparts. Thirdly, more hospital characteristics other than the number of hospital beds should be integrated with the current measurement of hospital attractiveness for predicting patient’s travel patterns. The reasons for some patients’ long distance travels to certain hospitals may be attributable to their healthcare plans that do not cover local hospitals. In this case, some interventions need to be initiated to encourage healthcare plans to include local hospitals for patients’ convenience without compromising their quality of care. Fourthly, the interaction among the degree of morbidity, patients’ travel patterns, and hospital charges will be examined in future studies. Finally, future research should also consider outpatient ambulatory care, emergency department visits, and breakdowns on various inpatient visits to obtain a fuller spectrum of medical services provided by hospitals.
